# Metabolic changes underlying drug resistance in the multiple myeloma tumor microenvironment

**DOI:** 10.3389/fonc.2023.1155621

**Published:** 2023-04-06

**Authors:** María Matamala Montoya, Gijs J. J. van Slobbe, Jung-Chin Chang, Esther A. Zaal, Celia R. Berkers

**Affiliations:** ^1^Division Cell Biology, Metabolism & Cancer, Department Biomolecular Health Sciences, Faculty of Veterinary Medicine, Utrecht University, Utrecht, Netherlands; ^2^Biomolecular Mass Spectrometry and Proteomics, Bijvoet Center for Biomolecular Research and Utrecht Institute of Pharmaceutical Sciences, Utrecht University, Utrecht, Netherlands

**Keywords:** cancer metabolism, multiple myeloma, drug resistance, bone marrow stromal cell (BMSC), fluxomics, metabolomics, tumor microenvironment, Bortezomib

## Abstract

Multiple myeloma (MM) is characterized by the clonal expansion of malignant plasma cells in the bone marrow (BM). MM remains an incurable disease, with the majority of patients experiencing multiple relapses from different drugs. The MM tumor microenvironment (TME) and in particular bone-marrow stromal cells (BMSCs) play a crucial role in the development of drug resistance. Metabolic reprogramming is emerging as a hallmark of cancer that can potentially be exploited for cancer treatment. Recent studies show that metabolism is further adjusted in MM cells during the development of drug resistance. However, little is known about the role of BMSCs in inducing metabolic changes that are associated with drug resistance. In this Perspective, we summarize current knowledge concerning the metabolic reprogramming of MM, with a focus on those changes associated with drug resistance to the proteasome inhibitor Bortezomib (BTZ). In addition, we present proof-of-concept fluxomics (glucose isotope-tracing) and Seahorse data to show that co-culture of MM cells with BMSCs skews the metabolic phenotype of MM cells towards a drug-resistant phenotype, with increased oxidative phosphorylation (OXPHOS), serine synthesis pathway (SSP), TCA cycle and glutathione (GSH) synthesis. Given the crucial role of BMSCs in conveying drug resistance, insights into the metabolic interaction between MM and BMSCs may ultimately aid in the identification of novel metabolic targets that can be exploited for therapy.

## 1 Introduction

Multiple myeloma (MM) is an incurable B-cell neoplasm characterized by the clonal expansion of malignant plasma cells in the bone marrow (BM) (1). MM is the 2^nd^ most common hematological malignancy (2) and accounted for 2.1% of all cancer deaths in the USA in 2022 (3). Over the last decade therapeutic advances (4), including the proteasome inhibitor Bortezomib (BTZ, VELCADE®) (1, 5), led to improvement in overall MM survival (1). BTZ targets mainly the β5(i)/β1(i) subunits of the 26S proteosome (6), resulting in a cascade of events that include the unfolded protein response (UPR) and amino acid deprivation, ultimately leading to cell death (7–9). However, MM remains an incurable disease, with only 57.9% of MM patients reaching 5 years survival (2012–2018) (3) and ultimately most MM patients relapse after BTZ treatment (10, 11). MM cells can develop drug resistance *via* multifactorial mechanisms (4, 8). In case of BTZ resistance, adaptation mechanisms include alterations at the level of the proteosome (mutations in the proteosome binding pocket, reduction of the 19S proteosome subunit, up-regulation of proteasomal machinery), upregulation of heat-shock proteins, genetic changes, activation of the aggresome-autophagy pathway, interactions within the MM tumor microenvironment (TME) and metabolic alterations (6, 8, 12, 13).

Metabolic reprogramming is regarded as an emerging hallmark of cancer (14–16) and is a potential target for cancer treatment (17). Metabolic rewiring fulfils the higher requirements of cancer cells for energy, building blocks for biosynthetic pathways and helps to maintain redox balance. These metabolic changes can be driven by genetic alterations, but can also be induced by the TME (18) and support both metastasis (16) and drug resistance (19). Metabolic reprogramming is a key feature of MM (7, 20) and metabolism further changes during the development of BTZ resistance (20–27). However, the influence of the MM TME on metabolic reprogramming of MM cells and its effect on drug resistance is still poorly understood.

In this perspective, we will highlight the most prominent metabolic alterations in MM and their contribution to drug resistance. Next, we will describe the current knowledge on the metabolic interactions between the BM TME and MM cells. As a proof-of-concept, we present novel data linking MM metabolic alterations induced by bone-marrow stromal cells (BMSCs) to drug resistance. Finally, we highlight how future studies on MM TME metabolism in the context of drug resistance can open novel therapeutic avenues for (relapsed) MM.

## 2 Multiple myeloma metabolism and its involvement in drug resistance

### 2.1 Glycolysis, serine synthesis pathway and pentose phosphate pathway

Glycolysis encompasses the breakdown of glucose into pyruvate, generating ATP and NADH. Most cancers are highly dependent on glycolysis (14, 28) and characterized by an increased production of lactate from pyruvate in the presence of oxygen. This so-called Warburg effect (29, 30) entails a faster, but less productive ATP generation compared to oxidative phosphorylation (OXPHOS) (31, 32). In addition to energy production, glycolytic intermediates can branch off into the pentose phosphate pathway (PPP) or serine synthesis pathway (SSP) to support the biosynthetic needs of cancer cells (33–35).

**Figure 1:**
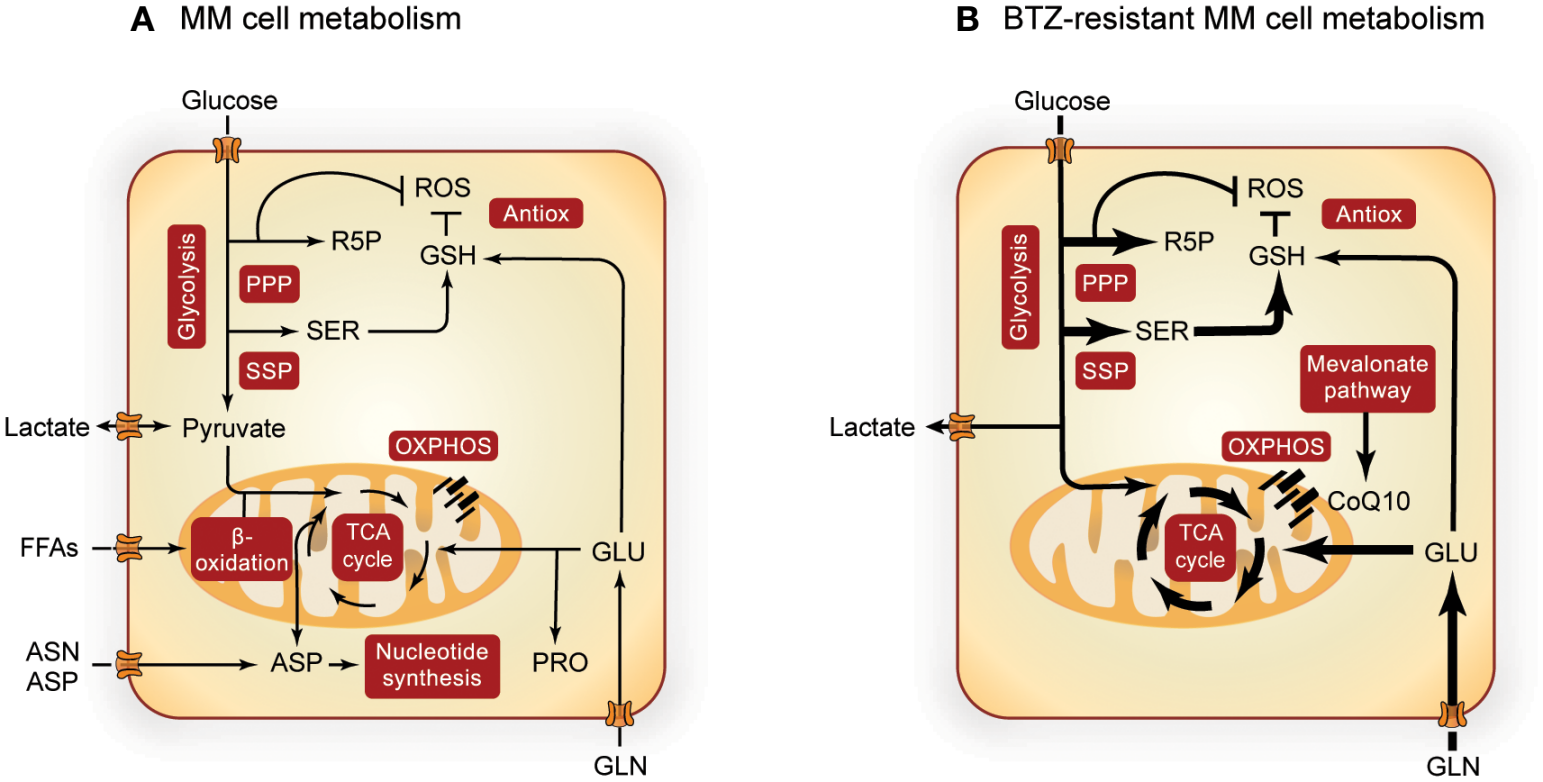
Overview of MM metabolism. Altered metabolic pathways in MM cells **(A)** and the most prominent metabolic alterations in BTZ-resistant MM cells **(B)**. Pathways involved in central carbon metabolism are presented in red boxes and width of the arrows indicate increased flux in BTZ-resistant cells. Metabolism of MM cells is further upregulated during BTZ resistance, with special importance in the SSP, PPP, TCA cycle, OXPHOS and GSH synthesis. PPP, pentose phosphate pathway; R5P, ribose 5-phosphate; ROS, reactive oxygen species; GSH, glutathione; Antiox, antioxidative response; SER, serine; FFAs, free fatty acids; ASN, asparagine; ASP, aspartate; GLU, glutamate; PRO, proline; OXPHOS, oxidative phosphorylation; CoQ10, coenzyme Q10; MM, multiple myeloma; BTZ, bortezomib.

Like most cancers, MM cells show an increased glycolic flux (**Figure 1A**) (19), which is sustained by increased expression of glucose transporters (GLUTs) (36) and glycolytic enzymes such as hexokinase 2 (HK2) (37, 38), phosphofructokinase (PFK) (39), pyruvate kinase M2 (PKM2) (40–42), pyruvate dehydrogenase kinase 1 (PDK1) (43, 44) and lactate dehydrogenase A (LDHA) (43, 45). The generated lactate is exported by monocarboxylate transporters (MCTs) (46–48) and promotes a pro-tumorigenic extracellular environment (49). In addition to glycolysis, both the PPP and SSP are upregulated in MM (**Figure 1A**). Glucose-6-phosphate dehydrogenase (G6PD), the rate limiting enzyme of the PPP, and its regulator protein disulfide isomerase family A member 3 pseudogene 1 (PDIA3P) are increased in MM patients (50). Notably, expression of glycolytic and PPP enzymes, such as LDHA, PDK1, PKM2, G6PD and PDIA3P, are also associated with poor prognosis (41, 51, 52) and low survival (50, 53) in MM patients. Furthermore, levels of 3-phosphoglycerate dehydrogenase (PHGDH), the rate-limiting enzyme of the SSP, were found to be increased in MM cell lines (54) and in MM patient cells compared to normal B-cells (35). In both plasma and BM of MM patients, serine levels decrease inversely proportional to the stage of the disease (55), which could be indicative of increased serine consumption as disease progresses.

When MM cells become resistant to BTZ, glycolysis (45, 56, 57), PPP (26, 50, 53) and SSP (26, 35, 54) are even further upregulated. This metabolic remodeling of BTZ-resistant MM cells (**Figure 1B**) is characterized by higher glucose uptake and lactate secretion (26, 45, 58), with upregulation of MCTs being related to low treatment response (59). Also hypoxic environment, a characteristic of MM tumors *in vivo*, increases the expression of several glycolytic enzymes (60) and has been related to drug resistance in MM (45, 56, 57). Furthermore, the SSP enzyme PHGDH is upregulated in MM cells from BTZ refractory patients and in BTZ-resistant cell lines (26, 35). In line with these findings, overexpression of PHGDH induces BTZ resistance and cell growth (35), whereas PHGDH inhibition or serine starvation enhance BTZ toxicity (26, 54). BTZ exposure is linked to the overproduction of reactive oxygen species (ROS), which triggers cell death. Since both PPP and SSP play important roles in the antioxidant response, these pathways are likely to counteract BTZ-induced oxidative stress (26, 35, 54, 58).

### 2.2 Mitochondrial energy metabolism and associated pathways

Pyruvate can be converted to acetyl-CoA and further oxidized in the mitochondrial tricarboxylic acid (TCA) cycle to generate reducing equivalents, which in turn are used for OXPHOS to produce ATP (61). Intermediates of the TCA cycle can also serve as building blocks for the biosynthesis of lipids and nucleotides. In addition to pyruvate, other sources can replenish the TCA cycle, such as fatty acids and amino acids, such as glutamine.

OXPHOS has been described as an important energy source for MM (21, 62, 63) (**Figure 1A**). Several studies show that high expression of mitochondrial enzymes of TCA cycle and OXPHOS are correlated with poor survival (21, 26, 58). MM cells mostly depend on glutamine to feed the TCA cycle (64, 65), supported by overexpression of Glutaminase-1 (GLS1) and glutamine transporters ASCT2, LAT1 and SNAT1 (65). Interfering with glutamine availability through inhibition of glutamine transporters or GLS or *via* glutamine starvation hampered cell viability in MM cells (64–66). In addition, lipid metabolism is emerging as an important pathway for MM proliferation. Acetyl-CoA synthetase 2 (ACSS2; involved in β-oxidation) expression is increased in MM patients (67) and inhibition of β-oxidation with Etomoxir (a CPT1 inhibitor) and/or Orlistat (a Fatty acid synthase (FASN) inhibitor), decreased MM proliferation (68). Furthermore, the membrane transporter fatty acid binding protein (FABP) (69, 70) as well as fatty acid import (55, 71) are also increased in MM. Support of biosynthetic pathways appears to be another important function of the TCA cycle. For example, inhibition of *de novo* pyrimidine synthesis from aspartate resulted in MM cell death (72). Pyrroline-5-carboxylate reductase 1 (PYCR1), involved in proline synthesis, showed increased expression in MM patients, which correlated with poor survival (73). In line, combinational treatment of BTZ and paragyline (PYCR1 inhibitor) showed a synergistic effect on MM (73).

OXPHOS and ATP production are especially high in BTZ-resistant cells (21, 62, 74) (**Figure 1B**). In addition, the mevalonate pathway is upregulated in BTZ-resistant MM cells, which generates the electron carrier coenzyme Q10 (CoQ10) and is thereby important for electron transport chain (ETC) function (21). This dependence of resistant MM cells on OXPHOS makes them susceptible to its inhibition. The mevalonate pathway inhibitor simvastatin lowered CoQ10 levels in BTZ-resistant cells, which is accompanied by decreased levels of TCA cycle metabolites and an enhanced BTZ-induced cell death both *in vivo* and *in vitro* (21). In line with these findings, the use of statins in MM patients is associated with reduced mortality (75) and lower levels of serum M protein, an indicator of MM remission (76). Glutamine addiction is a second signature that is further enhanced in BTZ resistance (21, 77) as is GOT1 expression (58). In line, GLS1 inhibition in MM cells resulted in a decrease of PI resistance (72), further underscoring the importance of glutaminolysis and associated biosynthetic pathways in MM drug resistance.

## 3 Metabolic interactions in the MM tumor microenvironment could enhance drug resistance

MM resides in a complex permissive niche of heterogeneous cells, forming the TME (78, 79), which is composed of cellular and non-cellular components (1, 20, 80) and plays a pivotal role in promoting tumorigenesis and drug resistance (81–84). Within the MM TME, bone-marrow stromal cells (BMSCs) are thought to be crucial in promoting MM drug resistance, which has been described to be induced through direct cell adhesion (84–90) and soluble factors (79, 91–94), as well as *via* different signaling pathways. BMSCs, especially fibroblasts, can be activated by soluble factors and turn into cancer-associated fibroblasts (CAFs). For example, it was observed that mesenchymal stromal cells (MSCs) express tumorigenic markers such as alpha smooth muscle actin (αSMA) when co-cultured with MM cells or by addition of MM-derived factors (95). In addition, αSMA expression was increased in BM and MSCs of resistant MM patients (96). The expression of fibroblast activation protein (FAP) increases in stromal cells after co-culture with MM cells (88) or with MM exosomes (97). Together, this suggests that the presence of MM cells induces a CAF-like phenotype in BMSCs.

It is known from other tumor types that cells in the TME can engage in metabolic crosstalk and cross-feeding with tumor cells in an adaptable manner and according to the tumor’s needs. For example, autophagy in CAFs has been described to feed cancer cells *via* the excretion of several amino acids, including proline, alanine or glutamine (98–102), and other fuel molecules such as fatty acids, ketone bodies, pyruvate and lactate (103, 104). In MM, autophagy in BM fibroblasts has been linked to drug resistance. Proteomics data indicate that upon BTZ exposure, BM fibroblasts from BTZ-resistant patients upregulate proteins and markers that are associated with cellular stress and autophagy (105). Autophagy in these fibroblasts is induced by TGFβ, a factor secreted by both BM fibroblasts and MM cells (106, 107) and inhibition of TGFβ could overcome BMSCs derived-BTZ resistance (105). Furthermore, bidirectional mitochondrial transfer can take place in direct contact between BMSCs and MM cells, enhancing mitochondrial activity and drug resistance in MM cells (82, 91, 108, 109). Recent reports also suggest that BMSCs can engage in metabolic crosstalk with MM cells. For example, glutamine demand in MM cells induced glutamine synthesis in the neighboring MSCs (110). MM-BMSCs exosomal crosstalk is positively regulated by an increased glutamate secretion and fine-tuned according to the metabolic demands (111). Such cross-talk will likely result in metabolic changes in MM cells. Moreover, many of the signaling pathways that are regulated by MM-BMSCs interactions are known to regulate downstream metabolic pathways (45, 112–119). However, little is known about the specific metabolic changes that occur in MM upon interaction with BMSCs and, importantly, how these changes contribute to the observed drug-resistance phenotype.

**Figure 2:**
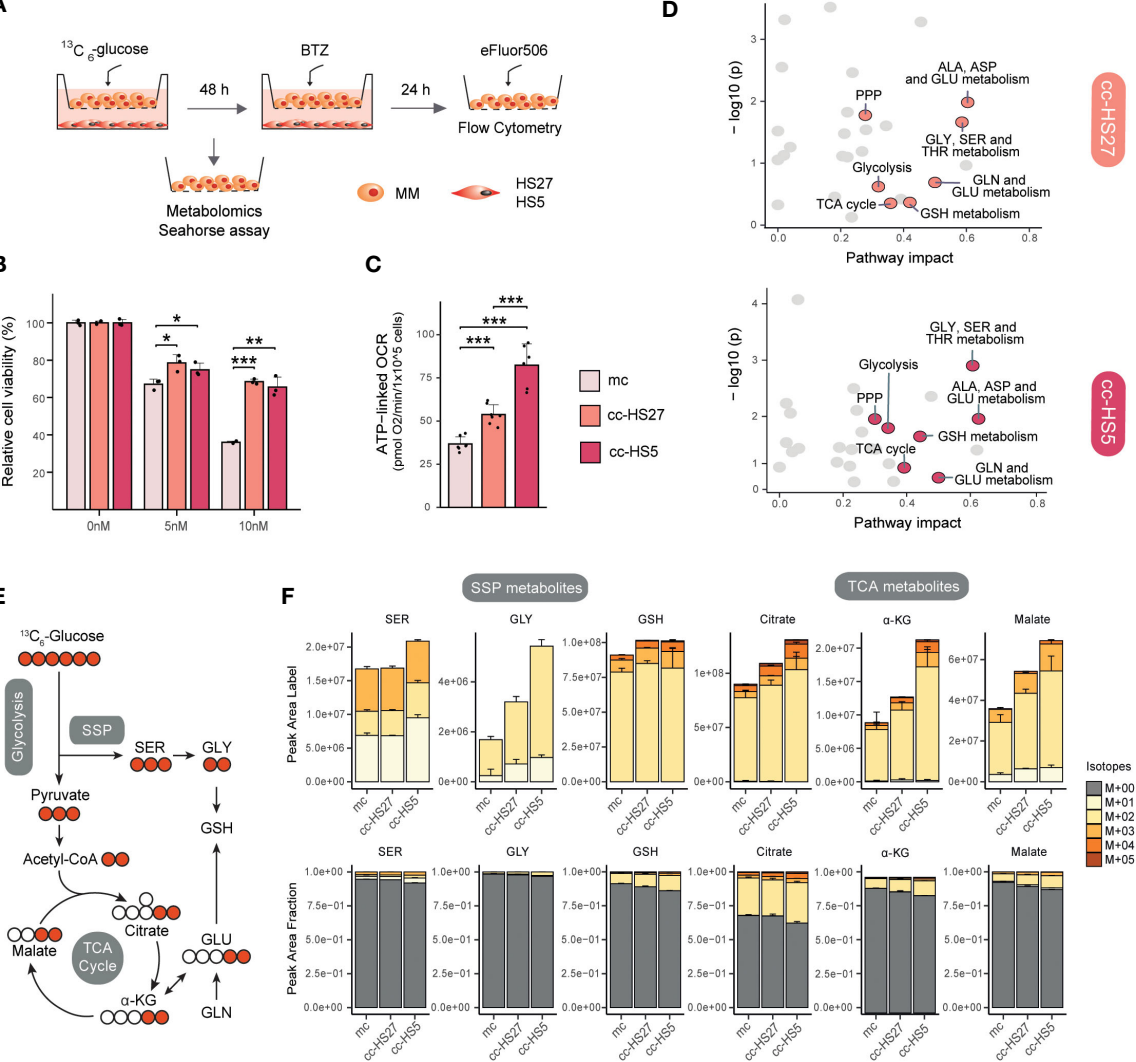
MM-BMSCs co-culture induces metabolic reprogramming and drug resistance-like metabolism. **(A)** Experimental layout for indirect co-culture of MM and BMSCs and overview of performed experiments. HS27a and HS5 human BMSCs were seeded in 12-well plates and allowed to attach overnight. Medium was replaced and RPMI8226 human MM cells were introduced in the upper chamber of Transwells® (TWs) (Corning, 0.4um, 12 well, polystyrene plates) with a seeding density of 1e^5^ cells/well, with a total volume of 3 mL/well. After 48h co-culture, MM cells were harvested for metabolomics or Seahorse assays or treated with Bortezomib (BTZ) for viability assays. For metabolomics experiments, media consisted of DMEM media containing 25 mM [U-^13^C]–glucose, 2mM glutamine and Penicillin Streptomycin. Metabolite extraction and LC-MS (pHILIC-QExactive) analysis were performed as described (26). For Seahorse experiment, MM cells were resuspended in Base DMEM Sigma D-5030 pH 7.4 supplemented with the same concentration of glucose, glutamine and Penicillin Streptomycin as metabolomics media and adding 5mM HEPES-NaOH and 21mM NaCl. After co-culture, MM cells were harvested and seeded and experiment was performed as previously described (21). For viability assays, different concentrations of BTZ (0, 5, 10 nM) were added for an additional 24h, after which MM cells were harvested, stained with the cell death die eFluor506 (BioScience; according to the manufacturer’s instructions) and then analyzed by flow cytometry (CytoFLEX). **(B)** Viability in MM cells after BTZ exposure in co-culture conditions: mc, mono-culture MM (light pink); cc-HS27a, co-culture MM with HS27a (orange); cc-HS5, co-culture MM with HS5 (dark pink). Data was analyzed with FlowJo™ and normalized to untreated cells (0 nM BTZ). Error bars depict SD of 3 independent wells from a representative experiment. **(C)** ATP-linked OCR of MM cells after co-culture conditions; mc: mono-culture MM (light pink); cc-HS27a: co-culture MM with HS27a (orange); cc-HS5: co-culture MM with HS5 (dark pink). The OCR was measured over time using the XFe24 Analyzer and ATP-linked OCR was calculated as the difference in OCR at basal conditions and after the addition of Oligomycin A. Error bars depict SD of 6-7 wells from a representative experiment. **(D)** Pathway enrichment analysis comparing mono- with BMSCs (HS5 or HS27a) co-cultured MM cells. Analysis was performed using Metaboanalyst package in R studio. A bigger pathway impact with smaller combined *p-*value (y-axis) is indicated as orange points (cc-HS27a) and dark pink points (cc-HS5) and it represents more reliably perturbed pathways in co-cultured vs. mono-cultured MM cells. **(E)** Schematic diagram of fluxomics, depicting the fate of ^13^C carbon into glycolysis, SSP, TCA cycle and GSH synthesis following [U-^13^C]–glucose uptake. Labeled and unlabeled C are represented with colored versus uncolored circles, respectively. **(F)** Metabolomic flux of [U-^13^C]–glucose into selected SSP and TCA cycle metabolites under mono- and co-culture conditions. Quantification of ^13^C-labeled peak areas (upper) and ^13^C-labeled fraction of total levels (lower) of serine (SER), glycine (GLY), glutathione (GSH), citrate, α-ketoglutarate (α-KG) and malate. Peaks were analyzed using TraceFinder software and isotopologue distribution was corrected for natural abundance of ^13^C. Data are presented as mean ± SD. Different colors represent the different isotopologues, whereby unlabeled metabolites (M+00) are grey, and ^13^C-labeled isotopologues are represented with yellow to red colors. Statistical significance was determined with TWO-way ANOVA. * *P* < 0.05, ** *P* < 0.01, *** *P* < 0.001. ALA, alanine; ASP, aspartate; GLU, glutamate; GLY, glycine; SER, serine; PPP, pentose phosphate pathway; TCA cycle, tricarboxylic acid cycle; α-KG, α-ketoglutarate; GLN, glutamine; GSH, glutathione; THR, threonine; BTZ, bortezomib; MM, multiple myeloma; mc, mono-culture MM; cc-HS27a, co-culture MM with HS27a; cc-HS5, co-culture MM with HS5; OCR, oxygen consumption rate.

To better understand the metabolic interactions between MM and BMSCs, we developed a non-direct co-culture system using BMSCs (HS5 and HS27a) and MM (RPMI8226) cell lines (**Figure 2A**). Flow cytometry-based cell viability assays confirmed that MM cells in co-culture become resistant to BTZ treatment, consistent with previous reports (120, 121) (**Figure 2B**). We next questioned whether metabolic changes induced in MM cells by BMSCs co-culture match those changes known to be involved in the development of BTZ resistance. Previous studies have shown that direct contact between MM and BMSCs increases mitochondrial metabolism as measured with oxygen consumption rate (OCR) (108, 109), which is also known to be important in BTZ resistance (21). Seahorse experiments in our system proved that indirect co-culture also significantly induces ATP-coupled OCR respiration (ATP-linked OCR), confirming the occurrence of a metabolic switch in MM cells upon co-culture with BMSCs (**Figure 2C**). To study this BMSCs-induced metabolic rewiring in more detail, MM cells were cultured alone or together with BMSCs in media containing [U-^13^C]–glucose to track glucose metabolism under these conditions (**Figure 2A**). Pathway enrichment analysis of total metabolite levels (i.e. the sum of all isotopologues) showed that BMSCs co-culture induces significant changes in MM cells (**Figure 2D**). Of note, amino acid metabolic pathways ranked amongst the most impacted pathways in co-cultured MM cells. Alanine, aspartate and glutamate metabolic pathways, which are linked to TCA cycle metabolism, were enriched in co-cultures with both HS27a and HS5 cells, whilst glycine and serine metabolism were significantly impacted in MM-HS5 co-cultures (**Figure 2D**). Additionally, PPP, TCA cycle and glutathione (GSH) metabolism were significantly upregulated in both co-cultures (**Figure 2D**). These data show that many pathways previously described to be of importance for BTZ-resistance are also significantly altered when MM cells are co-cultured with BMSCs.

To understand the contribution of glucose to these pathways, we analyzed the isotopologue distribution in SSP and TCA cycle metabolites (**Figures 2E, F**). When cells are cultured in [U-^13^C]–glucose, the SSP produces ^13^C_3_-serine (M+03). Serine (M+03) can be converted into glycine and subsequently into the antioxidative tripeptide GSH, both predominantly presenting the M+02 isotope (**Figure 2E**). Co-culture of MM cells with BMSCs, and particularly with HS5 cells, resulted in an increased synthesis of serine from glucose compared to MM mono-culture, as evidenced by higher absolute and fractional levels of ^13^C-labeled serine (**Figure 2F**). Downstream from serine, co-culture also increased the synthesis of glycine and GSH from [U-^13^C]–glucose (**Figure 2F**), as we also observed before in BTZ-resistant MM cells (26). In the TCA cycle, labelling from [U-^13^C]–glucose results in the formation of (predominantly) ^13^C_2_-citrate, -α-ketoglutarate and -malate (**Figure 2E**). Indeed, co-culture of MM with BMSCs increased both the levels of ^13^C-labelled TCA cycle metabolites, as well as the fractional contribution of glucose to this pathway, indicative of a higher glucose flux towards the TCA cycle (**Figure 2F**). Together, these results indicate that (metabolic) interactions between MM cells and BMSCs enhance SSP, TCA cycle and OXPHOS, which are key metabolic pathways in the adaptive response to BTZ.

## 4 Future prospects

Despite the interest in unveiling the crosstalk between MM and BMSCs, little is currently known about the metabolic interaction between these two cell types, especially in the context of drug resistance. So far, metabolic alterations related to BTZ resistance have mainly been studied in BTZ-resistant MM cell lines in mono-culture, in which drug resistance is induced by continuous drug exposure (122). Drug resistance in MM can be multiparametric, but the importance of the MM TME and especially BMSCs in driving drug resistance is widely accepted. In fact, recent studies showed the importance of targeting CAFs (123) and stroma interactions (124) to overcome drug resistance in MM. Here, we provide proof-of-concept data that demonstrate a previously unappreciated metabolic MM-BMSCs network in which indirect co-culture induces metabolic reprogramming and drug resistance-like metabolism in MM cells. This clear correlation between metabolic rewiring and BTZ resistance in the MM TME encourages further studies to determine the causal mechanism and further metabolic effects, which will benefit novel therapeutic paradigms, ultimately improving the treatment of relapsed MM.

First, to understand which metabolic vulnerabilities can be targeted in the MM TME, there is a need to unveil both commonly and differentially altered pathways induced by direct and indirect co-culture of MM with BMSCs. Most studies in terms of MM-BMSCs interaction have been performed in direct co-culture systems (86, 121), in which also metabolic alterations associated with mitochondria transfer have been reported (108, 109). Since cells in our co-culture system have no direct contact, our data point to a role for soluble factors in mediating MM metabolic rewiring. Indeed, several studies suggest that direct and indirect metabolic communication between MM and BMSCs could show common features and induce similar changes at the transcriptome and regulome level (115), as well as similar pathway activation (116, 117) and secretion of soluble factors (89).

A second open question is the precise mechanism underlying soluble factors-triggered metabolic changes in MM. Cytokines and growth factors released by MM cells or BMSCs can induce activation of metabolic enzymes, including the ones involved in antioxidant response and mitochondrial metabolism (60, 112, 125, 126), promoting drug resistance (127). Metabolites released by BMSCs may also directly feed into the metabolism of MM cells, as has been described in MM adipocytes (71, 128, 129). MSCs from MM patients showed increased glycolytic rate and lactate export compared to healthy donors (130). As (TME-derived) lactate can be used as a fuel for OXPHOS in MM cells (131), such metabolic cross-feeding could explain the higher ATP-linked OCR observed in MM cells under co-culture.

Finally, we here focused on energy metabolism and associated pathways, but many more metabolites are linked to MM drug resistance, including amino acids such as glutamate, proline (73, 113) or aspartate (72, 132). Lipid metabolism and β-oxidation have also been reported to be further enhanced in MM drug resistance (133–137), making interfering with lipid metabolism an interesting strategy to target resistance (68, 134). In addition, metabolites with immunosuppressant properties and that can also affect MM development are increased in MM in the context of the BM, including adenosine (138) and 2-deoxy-D-ribose (139, 140). Further elucidation of the BMSCs-induced metabolic rewiring in MM, including using different ^13^C/^15^N-labelled tracers and metabolomic and lipidomic approaches, will therefore likely unveil additional metabolic vulnerabilities in the MM TME.

In conclusion, this Perspective highlights the metabolic interactions within the TME that play a substantial role in drug response. Understanding this metabolic crosstalk will ultimately open new avenues to improve MM therapy.

## Data availability statement

The raw data supporting the conclusions of this article will be made available by the authors, without undue reservation.

## Author contributions

MMM, EZ and CB designed the project and experiments. MMM, JCC and GS performed experiments. EZ, JCC and MMM performed data analysis and visualization. MMM, CB and EZ wrote the manuscript. All authors contributed to the article and approved the final version.
